# Chemical metabolomics for investigating the protective effectiveness of *Acanthopanax senticosus* Harms leaf against acute promyelocytic leukemia†

**DOI:** 10.1039/c8ra01029c

**Published:** 2018-03-28

**Authors:** Yue Han, Ai-Hua Zhang, Ying-Zhi Zhang, Hui Sun, Xiang-Cai Meng, Xi-Jun Wang

**Affiliations:** Sino-America Chinmedomics Technology Collaboration Center, National TCM Key Laboratory of Serum Pharmacochemistry, Laboratory of Metabolomics, Department of Pharmaceutical Analysis, Heilongjiang University of Chinese Medicine Heping Road 24 Harbin 150040 China xijunwangls@126.com xijunwangtcm@126.com; State Key Laboratory of Quality Research in Chinese Medicine, Macau University of Science and Technology Avenida Wai Long, Taipa Macau China

## Abstract

Recent advances in the study of high-throughput metabolomics combined with high-resolution mass spectrometry have accelerated our understanding of the efficacy, mechanisms, and application of natural products. In this study, we have used chemical metabolomics to investigate and discover small molecule metabolites for the potential mechanism of *Acanthopanax senticosus* Harms leaf (ASL) against acute promyelocytic leukemia (APL). Based on high-throughput metabolomics, the underlying biomarker was found by combining chromatography coupled with quadrupole time-of-flight mass spectrometry with multivariate data analysis. The protective effect of ASL was dissected using biochemical indicators, pathology sections, immunohistochemistry, and multivariate analysis. Furthermore, 13 potential biomarkers associated with the pathway of sugar metabolism, amino-acid metabolism, nucleotide metabolism, and the metabolism of arachidonic acid were identified from serum samples. This study would help to understand chemical metabolomics for investigating the anti-APL effectiveness of ASL.

## Abstract 2

We have used chemical metabolomics to investigate and discover small molecule metabolites for the potential mechanism of *Acanthopanax senticosus* Harms leaf (ASL) against acute promyelocytic leukemia (APL).

## Graphical abstract

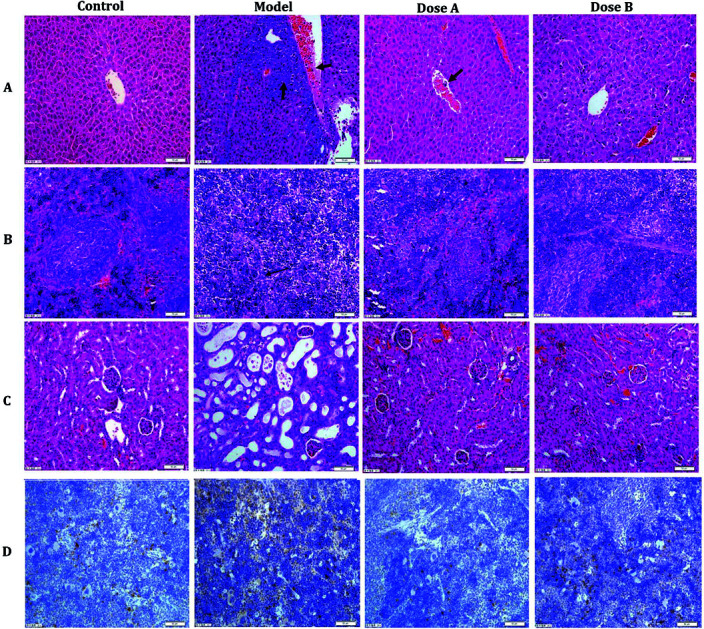

## 1 Introduction

Leukemia is a type of cancer that affects the blood and bone marrow. The white blood cells of the clone lose the ability to differentiate and remain in the state of cell development. There is a lot of white cell proliferation and gathering in the bone marrow and other locations. The result of proliferation and gathering of the white cells is the inhibition of hematopoiesis and the infiltration of tissue and organs. Acute leukemia and chronic leukemia are the two types of leukemia. Acute lymphatic leukemia (ALL) and acute myelocytic leukemia (AML) are two types of acute leukemia, which have different cell morphologies. APL with hemorrhage and blood coagulation dysfunction is a disease with high mortality.^1^

Anthracyclines, including daunorubicin and idarubicin, are the drugs used for APL treatment, but they are difficult to use alone due to their cardiac toxicity and the higher recurrence rate.^2^ All-trans retinoic acid is a medicine that can induce the change from promyelocytic to normal cells.^3,4^ Arsenic trioxide, which is a traditional Chinese medicine (TCM), also had a wonderful effect on APL.^5^ Combinations of all-trans retinoic acid and anthracyclines, arsenic trioxide and anthracyclines, and all-trans retinoic acid, arsenic trioxide, and anthracyclines have been widely used in clinical treatments.^6,7^ Caffeic acid and isofraxidin were found in *Acanthopanax senticosus* Harms, which is a typical folk medicinal herb that is widely distributed in the Northeastern part of China.^8,9^ In a previous study, ultra-performance liquid chromatography with mass spectrometry was used for the constituent analysis of extracts of the *Acanthopanax senticosus* Harms leaf (ASL).^10^ A total of 131 compounds were tentatively identified and 21 metabolites were identified *in vivo*.

Metabolomics has been proposed as a novel and comprehensive perspective for understanding and elucidating the etiology and mechanisms of complex biological systems.^11,12^ Recently, chemical metabolomics has been used to investigate the underlying action mechanisms and identify the potential biomarkers for accessing the action targets of TCM.^13–15^ Fortunately, a chemical metabolomics strategy, as an integrated, dynamic strategy, which is similar to TCM, has been rapidly developed. Here we focus on a metabolomic study concerning the potential biomarkers, the disorder of the metabolic pathways of APL and the therapeutic effect of ASL, which would be beneficial to understanding the action mechanism of the anti-APL effectiveness of ASL.

## 2 Materials and methods

### 2.1 Chemicals and reagents

Acetonitrile (HPLC grade) was purchased from Merck (USA). Ultra-pure distilled water was acquired from Watsons (China). Formic acid (HPLC grade) was bought from Kermel (China). Methyl alcohol and alcohol (95%) were purchased from Tianjin Fu Yu Fine Chemicals Co., Ltd. (China). Leucine enkephalin was obtained from Sigma (USA). The dried leaves of *Acanthopanax senticosus* Harms were purchased from Qitaihe city of Heilongjiang province. CD33 rabbit anti-human polyclonal antibody was obtained from Bioss (China).

### 2.2 Sample preparation

Fresh ASL was extracted in 50% ethanol in a ratio of 1 : 12 two times, each time for 2 h, and the ASL extract was filtered using a Buchner funnel with filter paper, condensed using a rotary evaporator at 50 °C, and freeze-dried into a powder. The freeze-dried powder was made into aqueous solutions with accurate concentrations of 4.212 g kg^−1^ and 6.318 g kg^−1^.

### 2.3 Animal handling

A total of 45 male NOD/SCID mice (18–20 g) were injected with HL-60 leukemia cells (5 × 10^6^) intravenously in the tail. These APL model mice (production license number: SCXK 2014-0001) and normal NOD/SCID mice (number: 15) were purchased from the Beijing Vitalstar Biotechnology Co., Ltd. Groups of five animals were fed in a mouse cage with a laminar flow rack in an SPF experiment lab with a room temperature of 22 ± 3 °C, relative humidity of 55 ± 5%, and a change every 12 hours between light and dark conditions. After a week, all animals were divided into four groups: control group, model group, ASL dose group (A), and ASL dose group (B). A total of 15 normal NOD/SCID mice and 15 APL NOD/SCID model mice were included in the control group and the model group, and were administered distilled water (0.1 ml/10 g) each day for 30 consecutive days. A total of 30 APL NOD/SCID model mice were average divided into two intervention groups. The doses of the ASL extract were set to 4.212 g kg^−1^ (dose A) and 6.318 g kg^−1^ (dose B). The extract (0.1 ml/10 g) was orally administered each day for 30 consecutive days. The experimental procedures were approved by the Animal Care and Ethics Committee at Heilongjiang University of Chinese Medicine and all experiments were performed in accordance with the declaration of Helsinki.

### 2.4 Serum sample preparation

Blood was collected from the eyeballs of the mice. The blood was put into a plastic pipe, stood for 30 minutes, and centrifuged at 3000 rpm (15 min, 4 °C). The supernatant of the serum was taken. To analyze the sample of serum metabolite, a total of 0.2 ml of serum was mixed with 1 ml of acetonitrile. The sample was vortexed for 5 min, stood for 30 min in ice, and then centrifuged at 13 000 rpm (15 min, 4 °C). The supernatant of the serum was taken, and blow-dried in nitrogen (40 °C). All dried samples were re-dissolved using 0.2 ml of acetonitrile, vortexed for 2 min, centrifuged at 13 000 rpm (15 min, 4 °C), and then the supernatant was taken and filtered using a 0.22 μm membrane. Finally, 3 μl of the serum sample was injected into the ultra-high performance liquid chromatography tandem mass spectrometry system for metabolomics analysis.

### 2.5 Pathology

The liver, spleen, and kidney were obtained and fixed using paraformaldehyde (4%), and the animal tissues were made into paraffin sections. These paraffin sections of tissue underwent xylene dewaxing twice (5 min), and then were put into absolute ethyl alcohol (5 min), 95% alcohol (2 min), 80% alcohol (2 min), 70% alcohol (2 min), and water (2 min). After a 5 min dye in hematoxylin, the paraffin section was washed using water. The slices were put into 1% hydrochloric acid alcohol (30 s) and soaked using water (15 min). After a 1 min dye in eosin, the paraffin section was washed using water and then soaked using water (5 min). Finally the paraffin section was dehydrated using a solution of 95% alcohol (1 min) twice and absolute ethyl alcohol (1 min) twice. We used the xylene to make the section transparent and sealed the section with neutral resins before histopathological changes were observed using an optical microscope.

### 2.6 Immunohistochemistry

The spleen was obtained from the NOD/SCID mice and fixed using paraformaldehyde (4%), and then the tissue of the spleen was made into paraffin sections. The sections of spleen were treated with poly-l-lysine and put into a roaster at 60 °C (5 h) for the close adhesion of the section. Then the sections of spleen were treated with xylene (80%, 90%, 100%) and ethanol (80%, 90%, 100%) for deparaffinization and rehydration. In order to inactivate the activity of the endogenous enzymes, the sections were put into 3% hydrogen peroxide at 37 °C (10 min), and washed thrice using water. In order to repair the antigen, the sections were immersed in 0.01 M PBS (pH 7.4) and boiled twice using a microwave oven. After putting 5% BSA in the section at 37 °C for 30 min, the section with CD33 antibody (1 : 500) was incubated overnight at 4 °C. The section was washed using 0.01 M PBS, and then was incubated for 30 min at 37 °C with a biotinylated secondary antibody. Finally, the section was rinsed and treated with diaminobenzidine (DBA) and hematoxylin.

### 2.7 Metabolomics

#### 2.7.1 Mass spectrometer conditions

The profiling of the serum sample was obtained using a Waters Acquity™ ultra-high performance liquid chromatography system with a MassLynx V4.1 workstation and an ACQUITY UPLC™ HSS T3 chromatographic column (100 mm × 2.1 mm, i.d., 1.7 μm). The column oven temperature was maintained at 45 °C. The flow rate and injection volume were set to 0.5 ml min^−1^ and 3 μl, respectively. The mobile phases were A (acetonitrile with 0.1% formic acid) and B (water with 0.1% formic acid). The following gradient eluting conditions were used: 0–1 min, 5–20% A; 6–7 min, 20–35% A; 7–9 min, 43–50% A; 9–16 min, 50–74% A; 16–19 min, 74–99% A.

The mass spectrometry conditions are listed as follows: the capillary voltage, sampling cone voltage, and extraction cone voltage for the positive mode were set to 2.8 kV, 45 V, and 4.0 V, respectively. The temperatures of the desolvation and the source for the positive mode were set to 250 °C and 110 °C, respectively. The desolvation gas flow for the positive mode was maintained at 500 l h^−1^. In the negative mode, the capillary voltage, the voltage of the sampling cone, and the extraction cone voltage were 2.0 kV, 45 V, and 4.0 V, respectively, the temperature of the desolvation and the gas flow for the desolvation were set to 350 °C and 700 l h^−1^, respectively, and the temperature of the source was 110 °C. All data were acquired in the centroid model from 50 to 1000 Da using Masslynx™ software. In order to ensure the mass accuracy and reproducibility, a solution of leucine enkephalin for [M + H]^+^ (*m*/*z* 556.2771) and [M − H]^−^ (*m*/*z* 554.2615) was used as a lock-mass for the ESI^+^ and ESI^−^ models, respectively. A QC sample was obtained per 10 samples of serum after the instrument was calibrated, ensuring the stability of the LC-MS system.

#### 2.7.2 Multivariate data analysis of metabolite profiling

UPLC-Q-TOF-HDMS was used for the discovery of potential biomarkers in the positive and negative ion modes. After all raw data was imported into the software MakerLynx V4.1, the calibration of retention time and mass number and the peak detection were carried out. The data matrix we obtained from MakerLynx V4.1 was imported into the software EZinfo 2.0, and disposed using the method of normalization and pareto scaling. Furthermore, the OPLS-DA analysis result from EZinfo 2.0 was used to obtain the potential biomarkers. Finally, the specific metabolites were identified using the element information comparison of the mass spectra using the software. The pathway analysis of specific metabolites was obtained using the web of MetaboAnalyst (http://www.metaboanalyst.ca) based on the database of KEGG and HMDB.

### 2.8 Statistical analysis

An unknown trend was discovered using a method of unsupervised multivariate statistical analysis in the treated groups, based on PCA. A 2-tailed, 2-sample Student’s *t*-test was used to test the mean values of statistically significant differences. The result (*P* < 0.05) was considered statistically significant. Finally, the resultant data matrices were mean-centered and pareto scaled for the multivariate analysis.

## 3 Results

### 3.1 The biochemical indicators

Compared to the control group, the white blood cell counts of peripheral blood in the model group are significantly higher. After a month of treatment, the white blood cell counts of peripheral blood were significantly reduced compared with those of the model group. However, the percentage of neutrophils and lymphocytes had no significant difference among the control group, model group, and two dose groups. The results are shown in the ESI, Table 1.†

### 3.2 The pathology observations and immunohistochemistry analysis

**Fig. 1:**
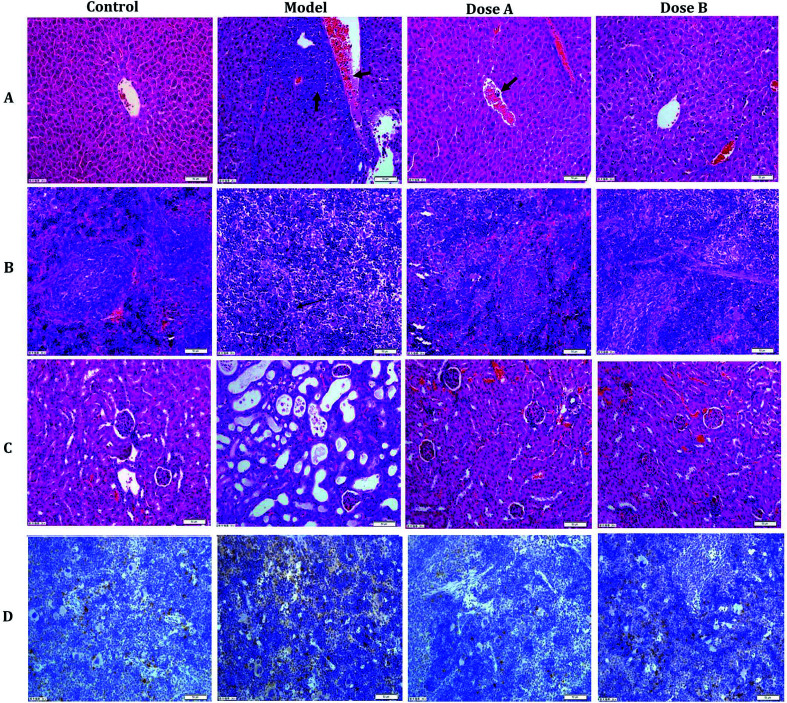
The pathology observations and immunohistochemistry analysis. Typical pictures of liver (A), spleen (B), and kidney (C) sections stained with H&E (200×). The image in (D) is the immunohistochemistry result (spleen).

After an interventional treatment of 30 days, the pathological results of the model group, normal group and dose group were observed using optical microscopy. The normal hepatocyte had a radial cord structure, but the hepatocyte cord of the model group developed a disordered arrangement. In addition, leukemic infiltration was found in the hepatic lobule. The organizational structure of each dose group mouse was repaired (Fig. 1A). Compared with the model group, the dose group and the normal group had a relatively complete organizational structure of the spleen. In the dose group, leukemic infiltration under the capsule of the spleen had an obvious decrease compared with that of the model group (Fig. 1B). As can be seen from the pathology result of the kidney that is shown in Fig. 1C, there were some inflammatory cell infiltrates and partial renal tubule degeneration in the model group. Compared with the model group, the degree of pathological changes and inflammatory cell infiltrates was reduced in the kidney of the dose group.

The immunohistochemistry result shows the brown granules of CD33 in the cytoplasm and interstitial. Compared with the model group, the positive expression rate of CD33 in the spleen of the dose group was significantly decreased (Fig. 1D and ESI, Table 2†).

### 3.3 Biomarker identification and metabolic pathway analysis

**Fig. 2:**
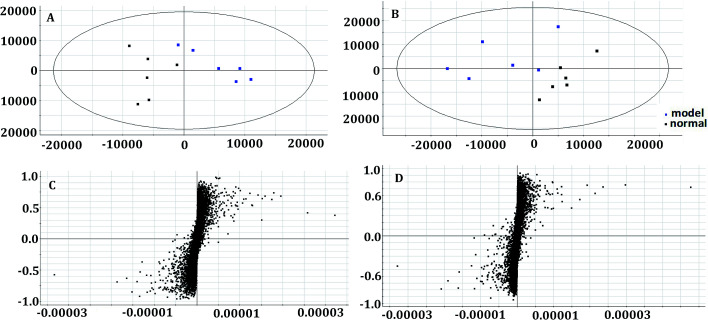
Multivariate analysis of plasma samples in the control group and model group in the positive mode ((A) the score plots of principal component analysis) and the negative mode ((B) the score plots of principal component analysis). The S-plot of OPLS-DA of APL in the positive mode (C) and negative mode (D).

**Fig. 3:**
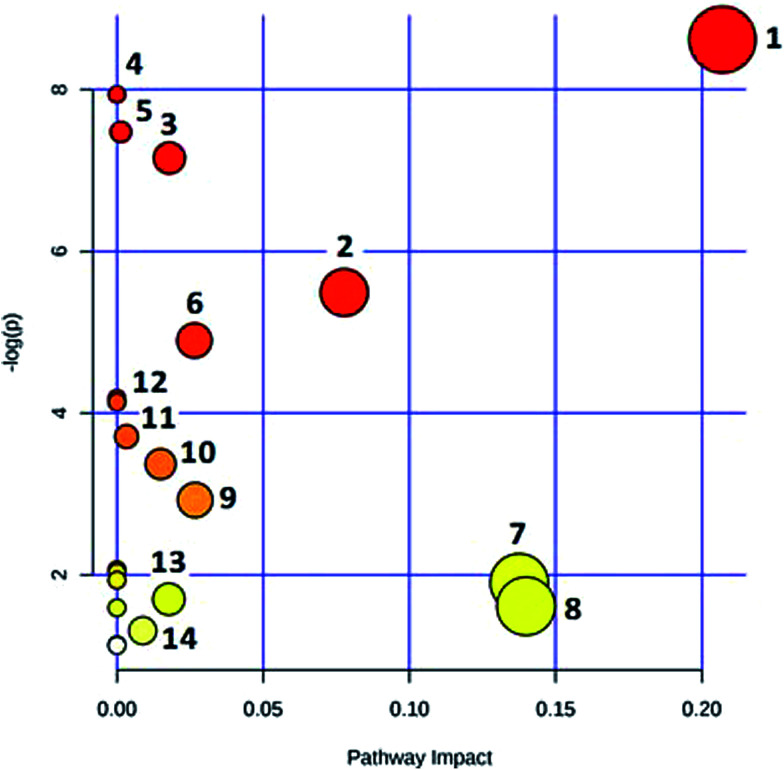
MetPA metabolic pathway analysis: (1) alanine, aspartate and glutamate metabolism; (2) citrate cycle (TCA cycle); (3) purine metabolism; (4) aminoacyl-tRNA biosynthesis; (5) propanoate metabolism; (6) valine, leucine and isoleucine biosynthesis; (7) pyruvate metabolism; (8) histidine metabolism; (9) d-glutamine and d-glutamate metabolism; (10) pyrimidine metabolism; (11) glycolysis or gluconeogenesis; (12) valine, leucine and isoleucine degradation; (13) butanoate metabolism; (14) arachidonic acid metabolism.

In the OPLS-DA model (Fig. 2C and D), the ion would have a greater VIP contribution for the cluster if the ion is far away from the origin. These farthest ions (VIP > 1) of the metabolites may be the potential biomarkers. At first, the study obtained the mass-to-charge ratio from the list of the OPLS-DA. Furthermore, a lot of molecular formulae which had an error below 10 ppm were obtained using the MetaboLynx 4.1 software. The possible structures and names of metabolites could be searched using a lot of databases (Chemspider, Human Metabolome Database, Metlin, and KEGG). A total of 48 ions were identified through the MS information, MS/MS fragmentation information and literature (ESI, Table 3†). 13 of the biomarkers identified, including l-glutamine, citric acid, *N*-carbamoyl-l-aspartic acid, histidine, isoleucine, hypoxanthine, valine, inosine, adenine, succinic acid, lactic acid, 5-OXO-ETE, and LTB4, were identified as having a greater contribution to clustering. Finally, the analysis of metabolic pathways was completed using KEGG and MetaboAnalyst software. The metabolic pathways related to disease are listed in Fig. 3 and in the ESI, Table 4.†

### 3.4 The protective effectiveness of ASL against APL

**Fig. 4:**
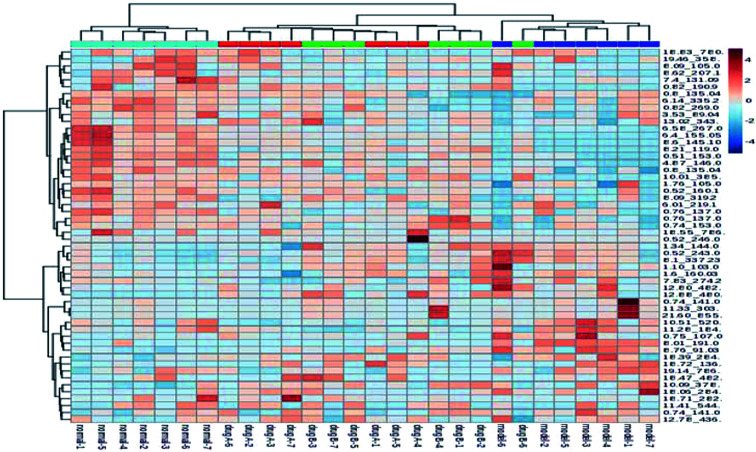
MetaboAnalyst analysis of protective effectiveness of ASL. Heatmap visualization of intervention effects of all doses on biomarkers (light blue: normal group, red: dose A group, green: dose B group, dark blue: model group).

**Fig. 5:**
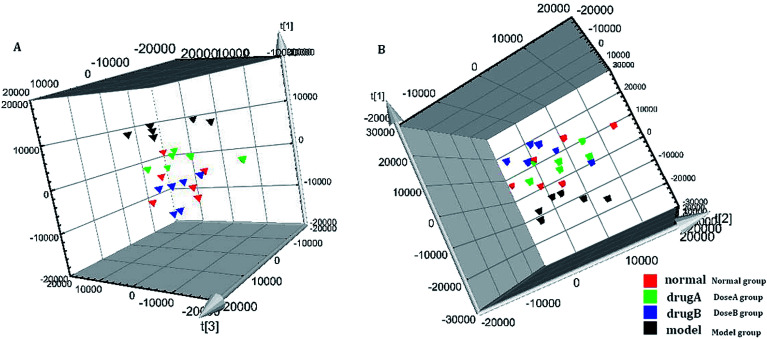
The tracking analysis of PCA score plots for APL after ASL treatment in the positive ion mode (A) and negative ion mode (B).

The metabolic profiles of the normal group, model group, and two dose groups were analyzed using the method of principal component analysis after administration for 30 days. For the two kinds of ion mode (Fig. 5), the score plots show an effect of separation between the control group and the model group. But the dose group had a closer vector distance with the control group. The heat map was generated using a MetaboAnalyst web for a more intuitive comparison, due to the APL in mice resulting in the disorder of endogenous metabolites. The expression of biomarkers in the model group was significantly different to that in the control group. Fig. 4 not only shows the significant differences in the model group and control group, but also the clustering of the two dose groups far away from the model group.

## 4 Discussion

NOD/SCID mice, which are immune deficient mice, are good animals for the experimental study of APL. CD33 is a differentiation antigen of the myeloid leukemia cell and has a higher expression in patients with acute myeloid leukemia.^16,17^ The higher expression of CD33 in the spleen of mice of the APL model showed that the model could be replicated. In the pathological section, the infiltration of cancer cells and the organizational structure change was observed in the image. In the supervised PCA mode of metabolomics, there was a significant distinction when comparing the control group with the model group. From all of these results, the replication of the APL model was proven to be successful.

In this study, UPLC-Q-TOF-MS combined with multivariate data analysis was used to find the potential biomarkers and metabolic pathway related to the disease, in order to discover the mechanism of acute promyelocytic leukemia on a metabolic level. The glucose metabolism disorder was an important mark in the cancer cell.^18,19^ Furthermore, the higher level of glycolysis could be induced by the resistance of leukemia cells.^20^ The level of glucose metabolism may be a prognostic factor in APL patients.^21^ In this study, the level of citric acid had a significant increase in the model group. The citric acid was an important reactant in the tricarboxylic acid cycle. The result illustrated that some amino acids and other substances may be transformed into citric acid, which makes up for the shortage of energy. The levels of glutamine, histidine, isoleucine, and valine were reduced in the model group, showing the disorder of energy metabolism. According to the literature, glutamine is an energy source in some proliferating cells such as cancer cells. All of these results may illustrate the disorder of energy metabolism in the model of AML.

At the primary stage of cancer, the replication stress of DNA due to the lack of nucleotides would lead to genetic instability.^22^ The metabolism of tumor cells was very strong, so the precursor material of purine was used significantly in the proliferation period compared with in the platform period.^23^ Therefore, there was a close relationship between the level of nucleotide and the occurrence and development of the tumor. In this study, the levels of aspartic acid and glutamine were reduced in the APL model. However, the aspartic acid and glutamine were the raw materials of purine nucleotides. The nucleotide metabolism and the energy metabolism may be disturbed in the APL mice.

According to a report, the biological metabolism of arachidonic acid being activated or blocked has great significance for the prevention and therapy of tumors.^24,25^ The activation of signal transduction pathways of MEK/ERK could inhibit apoptosis. Furthermore, the metabolites of arachidonic acid, including 5-HETE, 12-HETE, and TLB4, could promote the phosphorylation of MEK/ERK and inhibit the apoptosis of tumor cells.^26,27^ 5-OXE-ETE is a product of 5-HETE *via* the 5-lipoxygenase pathway, and could inhibit apoptosis of tumor cells *via* the 5-LO inhibitors.^28^ In this study, the levels of TLB4 and 5-OXE-ETE had a significant increase compared with those of the control group. In conclusion, the metabolism pathway of arachidonic acid may have been abnormal in the APL mice, so the apoptosis of tumor cells may be inhibited in the APL mice.

A total of 5 potential biomarkers including citric acid, lactic acid, l-glutamine, inosine, and isoleucine were adjusted to normal levels in the mice of the dose group. The ASL may have had a potential effect on APL by adjusting the disorder of some metabolic pathway nodes. Glutamine was an energy supply material for some proliferative cells, especially in the rapidly growing tumor cells.^29^ The glutamine was also the nitrogen source for the synthesis of purine and pyrimidine, maintaining the continuous proliferation of cells.^30^*In vitro* experiments showed that exogenous glutamine was depended on for survival and proliferation in cancer cells.^31,32^ In this study, the lower l-glutamine content was adjusted to a normal level in the ASL treatment group. The phenomenon showed a latent inhibition effect for the utilization of glutamine. Citric acid was an important reactant in the tricarboxylic acid cycle. Lactic acid was the end product of glycolysis. The amounts of citric acid and lactic acid had a trend of significant increase in the model group, illustrating the vigorous energy metabolism that took place in the APL model. Glutamine could provide the source of carbon for the biosynthesis of oxaloacetic acid, and then supplement the loss of citric acid due to the vigorous energy metabolism.^29^ Isoleucine could also be broken down into acetyl-CoA, and then enter the Krebs cycle for the energy supply. The amount of citric acid was increased, and the amounts of glutamine and isoleucine were decreased due to a relationship with the pathway of energy metabolism in the model group. Through the intervention of ASL, the citric acid, lactic acid, l-glutamine, and isoleucine were adjusted to normal levels, illustrating that ASL had a potential effect on the abnormal energy metabolism. At last, the intuitive analysis of PCA and the metabolomics heat map combined with biochemical indicators, pathology, and immunohistochemistry showed the potential effect on APL by treatment with ASL. The metabolomics strategy, as an integrated, dynamic strategy, which is similar to TCM, has been rapidly developed.^33–39^ This study also reveals that metabolomics provides an effective strategy for efficacy evaluation of TCM.

## 5 Conclusion

In this study, we established a serum metabolomics analytical method to discover the underlying metabolic biomarkers of APL and the protective effectiveness of ASL. A total of 48 differential metabolites were discovered and 13 of them had significant contributions. There was some disorder in the energy metabolism pathway, amino acid metabolic pathway, nucleotide metabolic pathway, and arachidonic acid metabolism associated with APL. After the intervention of ASL, citric acid, lactic acid, l-glutamine, inosine, and isoleucine were adjusted to the normal level. In conclusion, our investigation shows that chemical metabolomics was suitable for the evaluation of APL, and provided a promising strategy for efficacy evaluation of TCM.

## Conflicts of interest

The authors declare no competing financial interests.

## Supplementary Material

RA-008-C8RA01029C-s001
