# Supplementary material for: Chemical metabolomics for investigating the protective effectiveness of Acanthopanax senticosus Harms leaf against acute promyelocytic leukemia
Source: RSC Adv. 2018 Mar 28;8(22):11983–90. doi: 10.1039/c8ra01029c (PMC9079283; doi:10.1039/c8ra01029c)
Supplement: RA-008-C8RA01029C-s001 [file RA-008-C8RA01029C-s001.pdf]

Electronic Supplementary Material (ESI) for RSC Advances.
This journal is © The Royal Society of Chemistry 2018

Supplementary Table 1 The results of WBC, NEUT%, and LYMPH% of mice in different group (x±s) (n=6)

Group	Normal group	Model group	Dose group(A)	Dose group(B)
WBC(*109/L)	1.17±0.38	3.11±0.63※※	1.80±0.43△	2.78±1.48
NEUT (%)	59.79±16.63	61.71±0.82	59.48±20.14	64.51±18.41
LYMPH (%)	25.67±12.82	25.24±0.98	27.25±11.78	25.35±17.25

Model group compared with normal group，※※p<0.01; Dose group compared with model group, △△p<0.01, △p<0.05.

Supplementary Table 2 The results of the expression of CD33 in spleen of mice (x±s) (n=6)

Group	Normal group	Model group	Dose group(A)	Dose group(B)
CD33	0.0042±0.0008	0.0118±0.0050※※	0.0028±0.0014△△	0.0052±0.0034△

Model group compared with normal group，※※p<0.01; Dose group compared with model group, △△p<0.01, △p<0.05.

Supplementary Table 3 Proposed metabolites identified in plasma metablome.

No.	Rt(min)	Measured m/z	ESI+/-	Error (ppm)	Formula	Identify	Vip	Changes in model against healthy controls
1	0.51	153.028	-	-3.9	$C_4H_6N_2O_3Na$	D-Asparagine	1.5	↓
2	0.52	243.061	-	5.8	$C_9H_{11}N_2O_6$	Uridine	1.5	↑
3	0.52	160.133	+	-7.5	$C_8H_{18}NO_2$	Pregabalin	2.4	↓
4	0.62	105.009	+	-2.1	$C_3H_5O_4$	Malonic acid	1.8	↓
5	0.74	141.041	+	0.7	$C_7H_6N_2Na$	2-(Methylthio)phenol	1.8	↑
6	0.75	107.048	+	4.7	C_5H_8ONa	1-Penten-5-one	1.1	↑
7	0.76	137.035	+	-3.4	$C_5H_5N_4O$	Hypoxanthine	8.7	↓
8	0.8	135.04	-	3.7	$C_4H_8O_5$	Threonate	3.8	↓
9	0.82	190.965	+	0	$C_7H_5O_2Cl_2$	2,4-Dichlorobenzoate	1.1	↓
10	0.82	269.089	+	-6.3	$C_9H_{17}O_9$	2(α-D-Mannosyl)-D-glycerate	1.7	↓

11	0.87	174.97	+	5.1	$C_4H_8S_3Na$	5-Sulfinylpyruvic acid	1.4	↓
12	0.92	191.0234	-	2.7	$C_6H_7O_7$	Citric acid	1.0	↑
13	1.11	207.089	+	6.3	$C_7H_{15}N_2O_3S$	Diethyl tartrate	1.3	↓
14	1.5	119.027	+	0.5	$C_4H_7O_4$	Succinic acid	1.5	↓
15	1.7	160.038	-	4.4	$C_9H_6NO_2$	4,6-Dihydroxyquinoline	1.1	↑
16	2.0	105.034	+	-8.6	C_4H_9OS	2,4-Pentadienal	2.1	↑
17	2.2	91.0336	+	2.3	$C_3H_7O_3$	Lactic acid	1.2	↑
18	3.53	89.0445	+	-0.5	$C_3H_8NO_2$	Alanine	1.9	↓
19	3.85	204.05	+	2	$C_6H_7N_5O_2Na$	8-Hydroxy-7-methylguanine	1.3	↓
20	4.87	146.0659	+	-1.4	$C_5H_{11}N_2O_3$	L-Glutamine	2.4	↓
21	5.67	117.1790	+	2	$C_5H_{12}NO_2$	valine	1.2	↓
22	6.06	319.225	+	-6.9	$C_{18}H_{32}O_3Na$	Dimorphecolic acid	1.1	↓
23	6.14	335.224	-	8.9	$C_{20}H_{31}O_4$	Leukotriene B4	2.9	↓
24	6.4	155.0569	+	-7.2	$C_6H_{10}N_3O_2$	histidine	1.5	↓
25	6.58	267.082	-	4.5	$C_{10}H_{11}N_4O_5$	Inosine	1.1	↓
26	7.02	175.024	-	-5.2	$C_5H_7N_2O_5$	N-Carbamoyl-L-aspartic acid	1.1	↓
27	7.4	131.0986	+	3.6	$C_6H_{14}NO_2$	Isoleucine	1.3	↓
28	7.57	255.212	+	-5.9	$C_{19}H_{27}$	18-Nor-4(19),8,11,15-abietatetraene	1.7	↓
29	7.59	317.211	-	-7.9	$C_{18}H_{30}O_3Na$	5-Oxo-ETE	2.8	↑
30	7.67	333.204	-	-1.5	$C_{18}H_{30}O_4Na$	13(S)-HpODE	8.9	↓
31	7.9	315.195	-	-5.1	$C_{20}H_{27}O_3$	15-deoxy-δ-12,14-PGJ2	2.1	↓
32	8.1	337.238	+	4.4	$C_{20}H_{33}O_4$	6-trans-12-epi-LTB4	3.4	↑
33	8.3	245.176	+	-1.3	$C_{12}H_{25}N_2O_3$	Leucyl-Isoleucine	1.2	↓
34	8.45	291.132	+	4.5	$C_{10}H_{19}N_4O_6$	Argininosuccinic acid	4.7	↓
35	8.6	145.101	+	4.8	$C_9H_{14}Na$	Santene	1. 6	↓
36	8.62	207.139	+	-3.9	$C_{13}H_{19}O_2$	Benzyl hexanoate	1.2	↓

37	8.7	209.151	+	6.7	$C_{13}H_{21}O_2$	Undecanoic acid	1.6	↓
38	8.8	123.049	+	3	$C_6H_7N_2O$	Isonicotineamide	1.3	↓
39	8.93	161.132	+	9.3	$C_7H_{17}N_2O_2$	N(6)-Methyllysine	1.7	↓
40	9.19	333.206	-	4.5	$C_{18}H_{30}O_4Na$	7S,8S-DiHODE	4.0	↓
41	10.01	385.398	-	7.5	$C_{27}H_{45}O$	Cholesterol	2.6	↓
42	10.09	378.238	-	-0.5	$C_{20}H_{32}N_3O_4$	Sphingosine-1-phosphate	5.3	↑
43	10.12	273.225	+	7.3	$C_{14}H_{29}N_2O_3$	N(6)-(Octanoyl)lysine	1.3	↓
44	10.51	380.255	-	-9.3	$C_{18}H_{39}NO_5P$	Sphinganine-phosphate	3.0	↑
45	11.33	303.242	-	3.4	$C_{20}H_{31}O_2$	Arachidonic Acid (peroxide free)	2.0	↑
46	12.78	436.288	-	-2.3	$C_{21}H_{43}NO_6P$	PE(P-16:0/0:0)	2.2	↑
47	18.72	136.052	+	-7.1	$C_5H_6N_5$	Adenine	2.8	↑
48	19.02	136.021	+	-0.7	C_7H_6NS	Benzothiazole	3. 6	↑

Note: "↑" represents a trend of higher; "↓" represents a trend of lower

Supplementary Table 4 Result from ingenuity pathway analysis with Metabolyst based on KEGG

Pathway name	p	-log(p)	Holm p	FDR	Impact
1 Alanine, aspartate and glutamate metabolism	1.8081E-4	8.6181	0.014465	0.018681	0.20703
2 Citrate cycle (TCA cycle)	0.0041197	5.492	0.3131	0.077512	0.07773
3 Purine metabolism	7.8213E-4	7.1535	0.060224	0.021938	0.01793
4 Aminoacyl-tRNA biosynthesis	3.563E-4	7.9397	0.028148	0.019454	0.0
5 Propanoate metabolism	5.6681E-4	7.4755	0.056902	0.019454	0.00134
6 Valine, leucine and isoleucine biosynthesis	0.0074642	4.8976	0.65704	0.11681	0.0265
7 Pyruvate metabolism	0.034351	3.3711	1.0	0.85366	0.13756
8 Histidine metabolism	0.19901	1.6144	1.0	0.9691	0.13988
9 D-Glutamine and D-glutamate metabolism	0.053602	2.9262	1.0	0.42144	0.02674
10 Pyrimidine metabolism	0.034351	3.3711	1.0	0.31965	0.01492

11 Glycolysis or Gluconeogenesis	0.024442	1.9354	1.0	0.85366	0.0
12 Valine, leucine and isoleucine degradation	0.016	4.1352	1.0	0.18712	0.0
13 Butanoate metabolism	0.18254	1.7008	1.0	0.9691	0.01774
14 Arachidonic acid metabolism	0.26939	1.3116	1.0	1.0	0.00881

Note: the p is the original p value calculated from the enrichment analysis; the Holm p is the p value adjusted by Holm–Bonferroni method; the FDR p is the p value adjusted using False Discovery Rate; the Impact is the pathway impact value calculated from pathway topology analysis.
